# Assessment of tannic acid and glutathione in 38% silver diamine fluoride for dentine stain reduction with antibacterial efficacy: An *in vitro* study

**DOI:** 10.6026/973206300222602

**Published:** 2026-04-30

**Authors:** Nihar P, Mohd Sajad, Manish A.T, Asmita Sonawane, Nazia Khan, Monica Gogia, Anukriti Kumari

**Affiliations:** 1 Department of Conservative Dentistry and Endodontics, Ckstheja Dental College, Tirupati, DR. N.T.R University of Health Sciences, Vijayawada, Andhra Pradesh, India; 2 Department of Conservative Dentistry and Endodontics, Maulana Azad Institute of Dental Sciences, Mirdard Marg, Bahadur Shah Zafar Marg, LNJP Colony, New Delhi, Delhi, India; 3 Department of Oral Pathology & Microbiology, Triveni Dental College, Bodri, Bilaspur, India; 4 Department of Conservative Dentistry and Endodontics, Yogita Dental college and hospital, Khed, Maharashtra, India; 5 Department of Clinical Microbiology, Basic Medical Science, College of Medicine, Majmaah University, Al-Majmaah, Riyadh, Saudi Arabia; 6 Department of Pedodontics and Preventive Dentistry, Karnavati School of Dentistry, Gandhinagar, Gujarat, India; 7 Department of Oral Medicine and Radiology, School of Dental Sciences, Sharda University, Greater Noida, Uttar Pradesh, India

**Keywords:** Antibacterial efficacy, glutathione, silver diamine fluoride (SDF), tannic acid, tooth discoloration

## Abstract

Silver diamine fluoride (38% SDF) is an effective agent for caries arrest, but its clinical use is limited by undesirable dentine staining. Therefore, it is of interest to evaluate the effect of tannic acid and glutathione as additives in SDF on stain reduction and antibacterial efficacy using standardized *in vitro* methods. Sixty dentine specimens were treated and assessed for color change (ΔE) and antimicrobial activity (CFU counts), showing that tannic acid significantly reduced staining while maintaining antibacterial efficacy comparable to SDF alone. Glutathione demonstrated moderate stain reduction but a relative decrease in antimicrobial performance. Thus, we show tannic acid as an effective SDF additive that reduces staining while preserving antimicrobial efficacy.

## Background:

Silver diamine fluoride (SDF) has emerged as a highly effective, minimally invasive therapeutic agent for the management of dental caries, particularly in pediatric, geriatric and medically compromised populations. Its ability to arrest carious lesions, inhibit demineralization and exert potent antimicrobial effects has made it a preferred alternative to conventional restorative approaches [1]. The mechanism of action of 38% SDF is primarily attributed to the synergistic effects of silver ions, which possess broad-spectrum antibacterial properties and fluoride ions, which promote remineralization and enhance resistance to acid dissolution. Numerous studies have demonstrated its efficacy against cariogenic microorganisms such as Streptococcus mutans and Lactobacillus species, thereby supporting its role in preventive and therapeutic dentistry [2]. Despite these advantages, a significant limitation of SDF application is the formation of a characteristic black stain on treated dentine surfaces. This discoloration results from the precipitation of silver phosphate and other metallic oxides following the interaction of silver ions with tooth structure and oral fluids [3]. While the staining indicates caries arrest, it presents an esthetic concern, particularly in anterior teeth, thereby limiting patient acceptance and clinical application in visible areas. Consequently, there has been growing interest in modifying SDF formulations or introducing adjunctive agents to mitigate staining without compromising its antimicrobial efficacy [4]. Among the various agents explored, tannic acid has gained attention due to its strong protein-precipitating and metal-chelating properties. Tannic acid, a naturally occurring polyphenol, can interact with dentinal collagen and silver ions to form stable complexes, potentially reducing the extent of discoloration [5]. Additionally, tannins exhibit intrinsic antimicrobial activity by disrupting bacterial cell walls and inhibiting enzymatic activity, which may complement the antibacterial action of SDF. These properties suggest that tannic acid could serve as a dual-function additive, addressing both esthetic and antimicrobial aspects [6]. Similarly, glutathione, a tripeptide composed of glutamine, cysteine and glycine, has been investigated for its antioxidant and reducing capabilities. Glutathione can interact with free silver ions, potentially preventing their oxidation and subsequent precipitation that leads to staining [7]. Furthermore, its ability to neutralize reactive oxygen species may influence the biochemical environment within carious lesions. However, there is concern that excessive interaction with silver ions could reduce the availability of free ions necessary for antimicrobial activity, thereby affecting the therapeutic efficacy of SDF [8]. Although both tannic acid and glutathione have shown promise individually in modifying SDF-induced staining, there is limited comparative evidence evaluating their relative effectiveness in balancing stain reduction and antimicrobial performance [9]. Most existing studies have focused on either esthetic outcomes or antibacterial properties in isolation, with few directly comparing these additives under standardized conditions [10]. Therefore, it is of interest to assess the comparative effectiveness of tannic acid and glutathione as additives in 38% silver diamine fluoride for dentine stain reduction and antibacterial efficacy.

## Methodology:

This *in vitro* comparative experimental study was conducted over a period of six months from September 2025 to February 2026 to evaluate the effect of tannic acid and glutathione as additives in 38% silver diamine fluoride on dentine stain reduction and antibacterial efficacy. A total of 60 freshly extracted human permanent molars and premolars free from cracks, restorations, developmental defects and structural damage were included in the study. Teeth extracted for orthodontic or periodontal reasons were collected after obtaining ethical approval and were stored in 0.1% thymol solution until use. The selected sample size of 60 teeth provided adequate power for comparative analysis and allowed equal distribution among the experimental groups. All teeth were cleaned of soft tissue debris and calculus using ultrasonic scalers and were rinsed thoroughly with distilled water. Standardized dentine specimens were prepared by sectioning the crowns to obtain flat dentine surfaces. Artificial carious lesions were created on these dentine surfaces using a demineralizing solution under controlled laboratory conditions. Efforts were made to maintain uniformity in specimen dimensions and lesion depth across all samples. Baseline color measurements of each specimen were recorded using a spectrophotometer according to the CIELAB color system prior to intervention. The specimens were randomly allocated into three groups of 20 each using a computer-generated randomization method. Group I served as the control group and received 38% silver diamine fluoride alone. Group II received 38% silver diamine fluoride combined with tannic acid, while Group III received 38% silver diamine fluoride combined with glutathione. For antibacterial assessment, each group was further divided into two subgroups of 10 specimens each for microbial evaluation. The modified solutions were freshly prepared before application and the concentrations of tannic acid and glutathione were standardized based on prior evidence and pilot optimization. A uniform amount of the respective solution was applied to each specimen using a micro-brush and was allowed to act for a standardized duration of 2-3 minutes. Following application, the specimens were stored in artificial saliva at 37°C. For stain assessment, post-treatment color measurements were recorded at 24 hours, 7 days and 14 days using the same spectrophotometer. The overall color difference (ΔE) was calculated to quantify the degree of staining. Lower ΔE values indicated reduced staining. For antibacterial evaluation, dentine specimens were inoculated with standardized bacterial suspensions adjusted to 0.5 McFarland standards and incubated under appropriate conditions. After treatment, bacterial survival was assessed using colony-forming unit (CFU) count analysis. Samples obtained from treated specimens were cultured on selective media and colonies were counted after incubation. Reduced CFU counts indicated higher antibacterial efficacy. The data obtained were analyzed using SPSS version 26.0. Mean and standard deviation were calculated for both color change and CFU values. Intergroup comparisons were performed using one-way ANOVA followed by post-hoc Tukey test for pairwise comparisons. Repeated measures ANOVA was used for intragroup comparison of color changes over time. A p-value of less than 0.05 was considered statistically significant.

## Results:

A total of 60 dentine specimens were analyzed across three groups. Both stain reduction (ΔE values) and antibacterial efficacy (CFU counts) were evaluated and compared statistically. There was no statistically significant difference in baseline color values among the groups (p > 0.05), indicating homogeneity before intervention (Table 1 - see PDF). A statistically significant difference in ΔE values was observed among groups at all-time intervals (p < 0.001), with group ii showing the least staining (Table 2 - see PDF). One-way ANOVA revealed a significant difference between groups (F = 56.24, p < 0.001). Post-hoc analysis showed that tannic acid significantly reduced staining compared to both control and glutathione groups (Table 3 - see PDF). Group I and II showed significantly lower CFU counts compared to Group III, indicating superior antibacterial efficacy (Table 4 - see PDF). ANOVA showed a statistically significant difference among groups (F = 48.67, p < 0.001). No significant difference was found between Group I and II (p > 0.05), while both were significantly more effective than Group III (Table 5 - see PDF). Figure 1 (see PDF) shows comparison of baseline CIELAB color parameters (L*, a*, b*) among the three study groups. Group I (SDF), Group II (SDF + Tannic Acid) and Group III (SDF + Glutathione) showed comparable mean values with no statistically significant difference (p = 0.842), indicating homogeneity of samples prior to intervention. Error bars represent standard deviation the control group (SDF alone) demonstrated the highest staining but strong antibacterial efficacy. The tannic acid group showed the least staining with comparable antibacterial activity to SDF, while the glutathione group exhibited moderate stain reduction but significantly reduced antibacterial efficacy. These findings suggest that tannic acid is a more effective additive for balancing esthetic and antimicrobial outcomes.

## Discussion:

The present study showed that adding tannic acid or glutathione to 38% silver diamine fluoride (SDF) reduced dentine discoloration compared with SDF alone, while their effects on antibacterial activity were not identical. Tannic acid produced the lowest mean ΔE values in our study, indicating superior stain reduction, whereas glutathione showed moderate stain reduction. However, antibacterial performance was strongest with SDF alone and remained close to that level with tannic acid, while glutathione showed comparatively higher residual CFU counts. Overall, these findings suggest that tannic acid offered a better balance between esthetic improvement and preservation of antimicrobial action. Our findings are broadly consistent with Asghar *et al.* (2026) [11] who reported that tannic acid- and glutathione-modified 38% SDF both significantly lowered dentine staining compared with unmodified SDF, with the 15% tannic acid formulation showing the lowest ΔE values. That study also found that increasing tannic acid reduced the zone of inhibition, whereas glutathione did not significantly alter it, indicating that stain minimization may occur at the expense of some antibacterial activity. Our results similarly support the superior stain-reducing effect of tannic acid, although in our dataset tannic acid still maintained acceptable antibacterial efficacy. The present results also agree with the earlier work of Asghar *et al.* (2022) [12] who demonstrated that incorporation of green capping agents significantly reduced SDF-mediated staining and that the anti-staining potential followed the order tannic acid > glutathione. This ranking closely mirrors our observations, where tannic acid performed better than glutathione in reducing discoloration. Our glutathione findings are in line with Karuna *et al.* (2023) [13] who concluded in an *in vivo* split-mouth study that mixing 20% glutathione with 38% SDF effectively reduced tooth discoloration without compromising caries-arresting efficiency. Although our study also found that glutathione improved color outcomes versus SDF alone, the antibacterial effect in our specimens appeared weaker than that seen with tannic acid, suggesting that the interaction may depend on formulation, substrate, or *in vitro* conditions. Similarly, Kamble *et al.* (2021) [14] found that both potassium iodide and glutathione reduced discoloration after 38% SDF application, while SDF alone caused the greatest color change. Their study supports our observation that glutathione is useful for stain control, even if it may not be the strongest option available. Regarding antimicrobial performance, our control SDF group showed the lowest bacterial counts, which is consistent with Mei *et al.* (2013) [15] who showed that 38% SDF significantly inhibited multispecies cariogenic biofilm formation on dentin and reduced CFU counts compared with controls. This supports the strong antibacterial benchmark seen in our SDF-only group and explains why any additive must be evaluated carefully to avoid reducing this core therapeutic advantage.

## Conclusion:

Tannic acid showed the most effective reduction in SDF-induced dentine staining while maintaining antibacterial efficacy comparable to conventional 38% SDF. Glutathione provided moderate stain reduction but showed comparatively reduced antimicrobial performance. Therefore, tannic acid appears to be a more suitable additive for improving the esthetic acceptability of SDF without significantly compromising its therapeutic benefits.
